# Supplementary figures and images for: CTLA-4 antibody-drug conjugate reveals autologous destruction of B-lymphocytes associated with regulatory T cell impairment
Source: eLife. 2023 Dec 21;12:RP87281. doi: 10.7554/eLife.87281 (PMC10735222; doi:10.7554/eLife.87281)

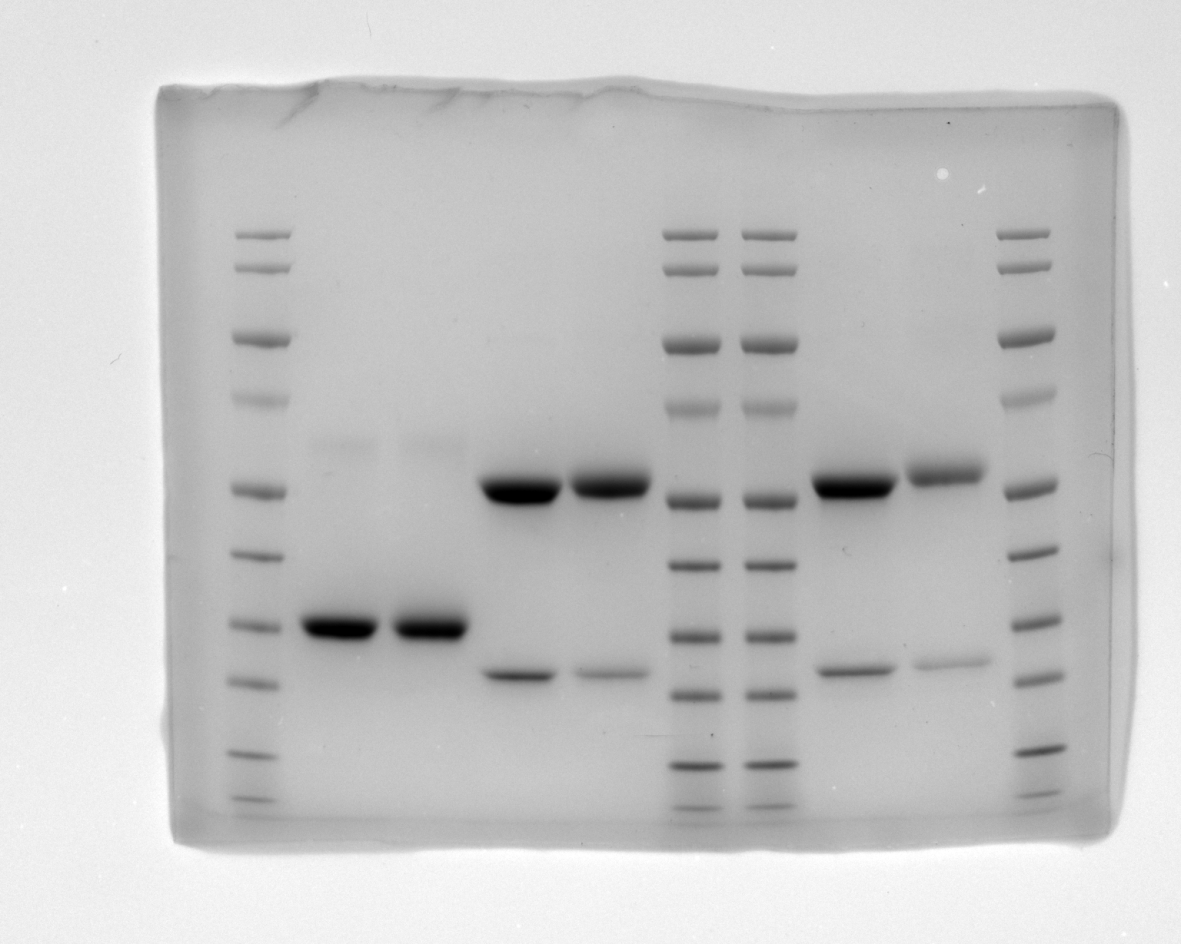

Supplement: Figure 1—source data 1. — Data 1. Panel A. SDS-gel; Data 2. Panel B. ELISA binding; Data 3. Panel C. Flow binding; Data 4. Panel E. Tregs; Data 5. Panel F. CTLA-4; Data 6. Panel G. Ki67; Data 7. Panel H. B cells; Data 8. Panel I. Ki67; Data 9. Panel J. Tregs; Data 10. Panel K. CTLA-4; Data 11. Panel L. Ki67; Data 12. Panel N. Mature B cells; Data 13. Panel O. Immature T1 B cells; Data 14. Panel Q. Progenitor B cells; Data 15. Panel R. CD21/CD35. [file elife-87281-fig1-data1.zip › Figure 1-Source Data/Figure 1- Source Data 1/PROTEIN_GEL_05012022_171323.tif]

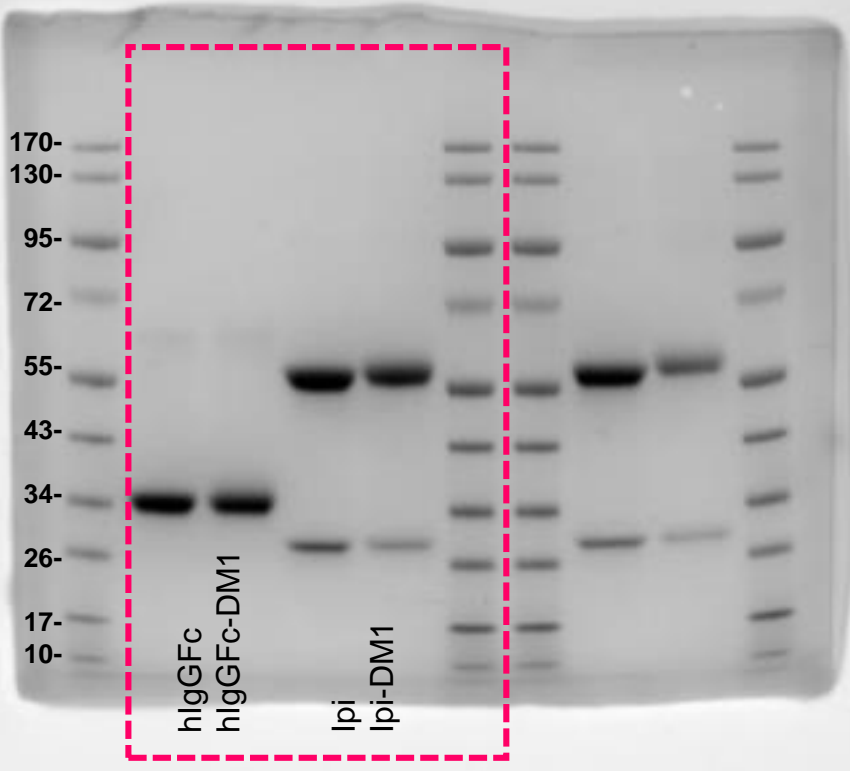

Supplement: Figure 1—source data 1. — Data 1. Panel A. SDS-gel; Data 2. Panel B. ELISA binding; Data 3. Panel C. Flow binding; Data 4. Panel E. Tregs; Data 5. Panel F. CTLA-4; Data 6. Panel G. Ki67; Data 7. Panel H. B cells; Data 8. Panel I. Ki67; Data 9. Panel J. Tregs; Data 10. Panel K. CTLA-4; Data 11. Panel L. Ki67; Data 12. Panel N. Mature B cells; Data 13. Panel O. Immature T1 B cells; Data 14. Panel Q. Progenitor B cells; Data 15. Panel R. CD21/CD35. [file elife-87281-fig1-data1.zip › Figure 1-Source Data/Figure 1- Source Data 1/SDS-reduced Gel processed.pdf]
