# Supplementary material for: CTLA-4 antibody-drug conjugate reveals autologous destruction of B-lymphocytes associated with regulatory T cell impairment
Source: eLife. 2023 Dec 21;12:RP87281. doi: 10.7554/eLife.87281 (PMC10735222; doi:10.7554/eLife.87281)
Supplement: Supplementary file 1. — Drug to antibody ratio (DAR). [file elife-87281-supp1.docx]

| Entry | *A*_280_ | *A*_252_ | CmAb (Moles) | CDM1(Moles) | DAR CDM1/CmAB |
| --- | --- | --- | --- | --- | --- |
| hIgGFC-DM1 | 0.51 | 0.48 | 6.92705E-06 | 1.1531E-05 | 1.7 |
| Ipilimumab-DM1 | 0.62 | 0.42 | 2.30582E-06 | 7.32032E-06  Drug to antibody ratio (DAR)= CDM1/ CmAB. DM1 known extinction coefficients (A_280_, 5700 M^-1^cm^-1^ ; A_252_, 28084 M^-1^cm^-1^ ). Experimentally derived extinction coefficients for Ipilimumab (A_280_, 242115 M^-1^cm^-1^ ; A_252_, 92989 M^-1^cm^-1^) and hIgGFc (A_280_, 64136 M^-1^cm^-1^ ; A_252_, 22544 M^-1^cm^-1^) | 3.2 |

**Table S1. Drug to antibody ratio (DAR)**
